# Home-Based Augmented Reality Exercise For People With Parkinson Disease: Qualitative Acceptability Study

**DOI:** 10.2196/70802

**Published:** 2025-10-06

**Authors:** Lotte E S Hardeman, Esther van Benten, Eva M Hoogendoorn, Maaike van Gameren, Jorik Nonnekes, Melvyn Roerdink, Daphne J Geerse

**Affiliations:** 1 Department of Human Movement Sciences Faculty of Behavioural and Movement Sciences Vrije Universiteit Amsterdam Amsterdam The Netherlands; 2 Institute for Movement Studies HU University of Applied Sciences Utrecht Utrecht The Netherlands; 3 Department of Rehabilitation, Centre of Expertise for Parkinson and Movement Disorders Donders Institute for Brain, Cognition and Behaviour Radboud University Medical Center Nijmegen The Netherlands; 4 Department of Rehabilitation Sint Maartenskliniek Nijmegen The Netherlands; 5 Department of Nutrition and Movement Sciences, NUTRIM Institute of Nutrition and Translational Research in Metabolism & MHeNs Institute of Mental Health and Neurosciences Faculty of Health, Medicine and Life Sciences Maastricht University Maastricht The Netherlands

**Keywords:** Parkinson disease, augmented reality, home-based rehabilitation, acceptability, physical therapy, neurorehabilitation

## Abstract

**Background:**

The rising prevalence of Parkinson disease and the growing demand on the health care system underscore the need for accessible and innovative care solutions, such as Reality Digital Therapeutics (Reality DTx)—an augmented reality neurorehabilitation program that delivers remotely prescribed gait and balance exercises for people with Parkinson disease to perform at home.

**Objective:**

At a preimplementation stage, this qualitative study aimed to explore the acceptability of Reality DTx.

**Methods:**

An exploratory qualitative study design was used. We conducted semistructured interviews, guided by the theoretical framework of acceptability, with 22 people with Parkinson disease who used Reality DTx at home for 6 weeks as part of a feasibility trial. We conducted a reflexive thematic analysis using an inductive, semantic approach informed by critical realism.

**Results:**

The results of the reflexive thematic analysis are described in 3 themes and 9 subthemes. The 3 themes are “there was considerable interindividual variation,” “the intervention is complementary to supervised physical therapy,” and “adherence in the long term is crucial.” Participants reported variable perceptions of effectiveness and variable experiences of effort to complete the Reality DTx program. They viewed Reality DTx as a valuable complement to supervised physical therapy and emphasized the indispensable role of the physical therapist for external control of long-term exercise adherence and for meaningful feedback on motor performance, as well as the desire for social connection. Flexibility in time and location was mentioned as a very important program characteristic, supporting long-term exercise adherence. Suggestions for improvement included enhanced visibility of progression in scores, increased variation in games, and the integration of competitive elements.

**Conclusions:**

Remotely prescribed, gamified, augmented reality exercises at home, complementary to supervised physical therapy, are acceptable to people with Parkinson disease. The findings inform future Reality DTx development and implementation from the perspective of people with Parkinson disease, which should be weighted with the perspectives of other stakeholders such as clinicians and other key decision-makers.

## Introduction

### Background

Parkinson disease is a neurodegenerative disease characterized by nonmotor symptoms, such as apathy and cognitive deficits, and motor symptoms, of which gait and balance impairments are already present early in the course of the disease, affecting everyday mobility and increasing the risk of falls [1,2]. The number of people with Parkinson disease is expected to exceed 13 million globally by 2040 [3]. This rise, along with the growing demand on the health care system, highlights the need to enhance access to treatment and improve the health care system’s efficiency. One way to improve accessibility for people with Parkinson disease is through technology-supported, home-based care [4-6]. This also accommodates the challenges people with Parkinson disease often face with rehabilitation, such as motor symptom fluctuations, lack of time, and poor accessibility to exercise locations or lack of transportation [7-9].

This study describes the acceptability of a particular digital therapeutic innovation for home-based care, namely Reality Digital Therapeutics (Reality DTx), an augmented reality (AR) neurorehabilitation program with gamified AR exercises specifically designed for people with Parkinson disease to train their gait and balance at home. With AR glasses, the real world is augmented with spatially aware digital objects, so-called holograms, while the real world remains visible through transparent see-through lenses. Besides providing gait and balance exercises, Reality DTx aims to increase therapy adherence by making rehabilitation accessible at home, available at any time, and enjoyable through gamification [10,11]. Our recent feasibility trial showed that, for people with Parkinson disease with modified Hoehn and Yahr (H&Y) disease severity 2 to 2.5, Reality DTx is feasible for home use, with a high adherence (ie, participants performed 104% of the prescribed exercise sessions and 88% of the prescribed exercise minutes over 6 weeks) [11]. It was further found to be safe, usable, and progressive but achievable (ie, increasing difficulty while maintaining high performance), and it showed promise to improve aspects of gait, balance, and fall risk [11]. This study, as part of the feasibility trial, explores the acceptability of Reality DTx for gait and balance exercises at home from the perspective of people with Parkinson disease.

Implementation of technological innovations in health care often fails [12,13]. One of the reasons is that the users’ motivations, values, and norms lead to resistance or rejection of the technology, or that technological adaptations over time do not meet users’ needs [12,13]. Therefore, early engagement of and regular evaluations with key stakeholders are crucial. In the case of Reality DTx, these key stakeholders include the innovation recipients (people with Parkinson disease), the innovation deliverers (the therapists), and the key decision-makers (physical therapy clinic owners, policy makers, and health care insurers) [14]. The acceptability of innovation among key users is one of the critical outcome measures to assess during development and implementation [15,16], yet an evaluation of acceptability is often lacking in technology health care research [6,12,13,17].

This study attempts to address this limitation by focusing on the acceptability of Reality DTx for gamified gait and balance exercises at home among people with Parkinson disease, the innovation recipients. They participated in the very first feasibility trial with AR gait and balance exercises at home [10,11]. The stage at which this study is conducted can thus be considered a preimplementation stage [15,18,19]. Acceptability is defined in line with the theoretical framework of acceptability (TFA) of health care interventions, which states that acceptability is “a multi-faceted construct that reflects the extent to which people delivering or receiving a healthcare intervention consider it to be appropriate, based on anticipated or experienced cognitive and emotional responses to the intervention” [16]. The TFA implies that acceptability should not solely be evaluated through commonly used measures of observed behavior, such as dropout and adherence rates, but must also include users’ knowledge and experiences with the health care intervention, as behavioral measures may not fully explain why participants’ withdrawal or adherence rates are low or high.

To illustrate this, 2 systematic reviews showed that home-based technology-enhanced interventions often used adherence logbooks or closed-ended satisfaction questionnaires to assess acceptability, with few studies exploring subjective user experiences underlying these numbers through qualitative evaluations [20,21]. The UK Medical Research Council guidance for the development and evaluation of complex interventions advocates for such qualitative or mixed methods process evaluations [22]. A qualitative process evaluation may help to identify intervention components, intervention outcomes, contextual factors, and their interactions. These links, so-called mechanisms of change, could provide insights into how interventions achieve their partially context-dependent effects. The Reality DTx program demonstrated feasibility and effectiveness in a research setting [11], but this may not translate to daily clinical practice due to contextual differences. In clinical practice, therapists, instead of researchers, provide personalized exercise prescriptions tailored to rehabilitation goals, which potentially enhances the experience for people with Parkinson disease. A qualitative evaluation could uncover the subjective mechanisms driving the high adherence and progressive-but-achievable character of the program shown in our feasibility trial [11]. Despite the growing number of studies on technology-based interventions for people with Parkinson disease, qualitative assessment of acceptability in this field is still limited, while the examples provided here highlight its relevance [23,24].

### Objectives

The aim of this qualitative study was to explore acceptability through in-depth direct experiences of people with Parkinson disease who received the 6-week Reality DTx gamified gait and balance program at home as part of our feasibility trial [10,11], complementary to more indirect quantitative acceptability indicators such as adherence and dropout rates. At this preimplementation stage, these direct responses of people with Parkinson disease could provide valuable insights into the mechanisms of change (eg, factors that motivate participants to train with Reality DTx) in the context of digitally supported independent exercise at home [6,16,18,22]. Therefore, this study adopted a qualitative approach using semistructured interviews. The perceptions and experiences of people with Parkinson disease will be discussed in relation to the current literature on technology-enhanced interventions. Recommendations for future development of Reality DTx and challenges for implementation are outlined. In a wider context, the study will provide insights into the experiences of people with Parkinson disease with technology-based rehabilitation at home that may stretch beyond the particular use case of Reality DTx in people with Parkinson disease.

## Methods

### Ethical Considerations

Ethics approval was obtained from the accredited Medical Research Ethics Committees United, the Netherlands (R22.076, NL82441.100.22). Participants provided written informed consent obtained by LESH, DJG, or EMH before participating in the feasibility study. The study was conducted and reported according to the Consolidated Criteria for Reporting Qualitative Research (COREQ) [25]. Consent has been obtained from all identifiable individuals included in this manuscript.

### Study Design

This exploratory qualitative study was guided by a critical realist epistemology, seeking to understand how participants experienced the Reality DTx exercise program in ways that reflect aspects of their lived reality, using semistructured interviews. This study was part of a larger feasibility study that evaluated the feasibility and potential efficacy of Reality DTx [10,11].

### The Reality DTx Program

**Figure 1:**
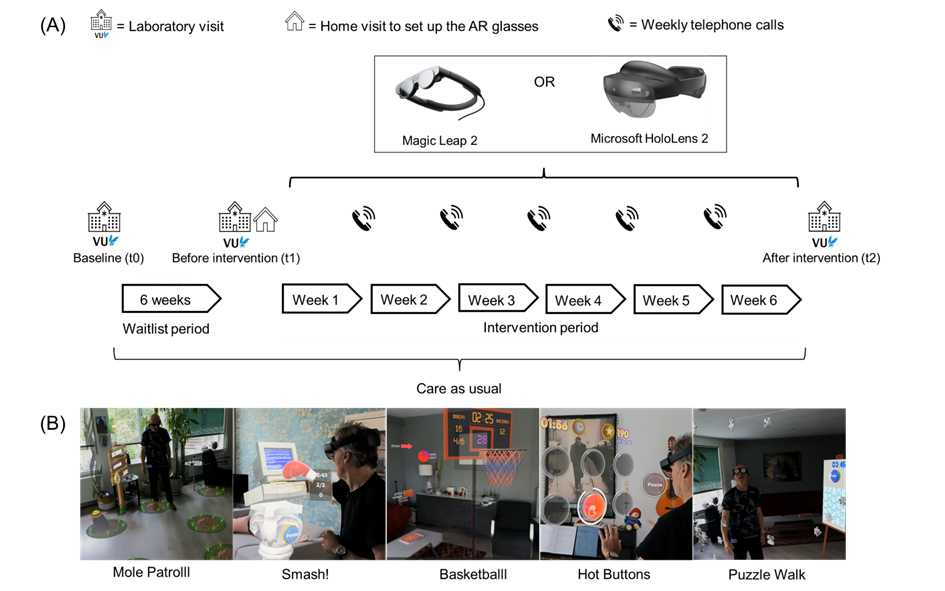
(A) Overview of the procedure of the waitlist-controlled feasibility trial, reprinted from the study by Hardeman et al [11]. (B) Images of the 5 Reality DTx exercises (from left to right): Mole Patrolll, Smash!, Basketball, Hot Buttons and Puzzle Walk.

The Reality DTx neurorehabilitation program is a class I CE-marked medical software app for AR glasses for home-based gait and balance exercises (Figure 1A). Following clinical guidelines [26-28], participants were instructed to use Reality DTx 6 weeks at home for at least 30 minutes per day and 5 days a week. Participants completed the 5 complementary AR exercises (every 3-min game was prescribed twice; Figure 1B), either in a single session or in so-called “exercise snacks” divided over the day. Among these 5 AR exercises, Mole Patrolll is a goal-directed walking exercise that targets gait initiation, dynamic balance, turning, stopping, and strength (in squat mode). The user is challenged to stomp on appearing moles before they disappear. Smash! is a boxing exercise that targets gait, dynamic balance, weight shifting, and turning. The user walks between 2 plinths and smashes as many items as possible from each. Hot buttons is a dynamic reaching exercise that targets functional reaching, reaction time, and dynamic balance. The user must press as many buttons as possible (1×3, 2×3, or 3×3 configurations) before they disappear. Basketball is a sit-to-stand exercise that targets dynamic balance and lower-limb muscle strength. The user completes sit-to-stand movements or squats and throws basketballs into the hoop. Puzzle walk is a goal-directed walking exercise that targets gait, dynamic balance, turning, stopping, and functional reaching. The user collects puzzle pieces at various heights and places them on the easel as fast as possible. The exercises increase in difficulty by reducing the appearance duration of items (eg, moles and buttons) or by increasing the number of items or required movements, such as the smash items on the pillars, puzzle pieces, or the number of sit-to-stands or squats. Alongside usual care, researchers trained the participants in using Reality DTx and prescribed and supervised the program through weekly calls to personalize the program’s frequency, difficulty, type of AR exercise, and/or duration using shared decision-making and with input from adherence and performance measures from the web portal [11]. Participants received feedback on game performance in the form of in-game and postgame scores and encouraging commentary from the AR glasses (the additional file in the study by Hardeman et al [10] provide more details on these scores and the AR exercises).

### Participants

Participants were recruited through convenience sampling, mainly via presentations at various Parkinson community groups hosted by the Dutch Parkinson Association in the west of the Netherlands, but also via physicians in our network and via the website of the Dutch Parkinson Association. Participants contacted the researchers voluntarily if they were interested.They were eligible to participate in the feasibility trial if they were diagnosed with Parkinson disease according to the UK Parkinson’s Disease Brain Bank criteria (H&Y 2-4) and if they experienced bothersome gait and/or balance impairments, based on self-report. Participants were excluded if they comply with the following criteria: (1) a sign of inability to comply with protocol, (2) additional neurological diseases and/or orthopedic problems seriously interfering with gait and balance function, (3) insufficient physical capacity or cognitive and/or communicative inability to understand instructions and participate in the tests (as observed by the researchers), (4) visual or hearing impairments (after corrective aids), (5) severe visual hallucinations or illusions, (6) an inability to walk independently for 30 minutes, and (7) no stable dosages of dopaminergic medication. There were no additional criteria, nor a minimum treatment dose, to be eligible to participate in the semistructured interviews. There were no restrictions to usual care. Eligibility criteria were checked through telephone screening before enrollment and again during the baseline (t0) laboratory assessment of the feasibility trial by researchers LESH, EMH, and DJG (further details on eligibility screening are given in the study by Hardeman et al [10]). Three laboratory assessments were conducted at t0, before intervention (t1), and after intervention (t2) to evaluate feasibility in terms of safety, adherence and user experience, and potential efficacy with key clinical gait and balance tests and a targeted walking-related fall risk assessment, performed by the researchers (Figure 1 [11]). All participants of the feasibility trial were characterized at t0 by collecting demographics; the Montreal Cognitive Assessment, the Movement Disorder Society—Unified Parkinson’s Disease Rating Scale (MDS-UDPRS), and the Physical Activity Scale for the Elderly. Following t2, all participants were invited to be interviewed. 92% (22/24) of the participants who started the Reality DTx program were interviewed. Two (8%) participants were not interviewed because they dropped out of the study in the first and fourth weeks of the intervention period due to too many technological issues with the AR glasses and due to medical circumstances unrelated to the study, respectively [11].

### Procedure

The interviews were conducted after the postintervention gait and balance laboratory assessments of the feasibility trial. At the time of the interview, participants had not yet received personal feedback on their results of the gait and balance and targeted walking-related fall risk assessments (Figure 1). The interviews took place in a quiet, private room at the Vrije universiteit Amsterdam on the assessment day or, based on the participants’ preference, via telephone on another day. Because some participants experienced difficulty in finding the right words to express themselves, 4 of the 8 partners present during the interview assisted in clarifying their views. These explanations were only used as such and were not included as primary data in the analysis.

The interview guide was carefully constructed with standardized topics and questions but allowed the researchers to spontaneously react to the participants’ responses with unscripted follow-up questions, as prescribed by reflexive thematic coding to ensure in-depth exploration [29]. The interview guide was partly based on the TFA [16], which consists of 7 acceptability indicators identified through an extensive literature search of health care interventions. These indicators include affective attitude, perceived effectiveness, intervention coherence, self-efficacy, opportunity costs, burden, and ethicality. We followed the operationalization of the TFA indicators by Gerards et al [30], specified in Table 1. Besides the TFA indicators, the interview guide included the following topics: (1) program-specific factors, such as the type of AR exercises, frequency, intensity, and duration; (2) context-specific factors, such as exercise at home and remote supervision; (3) the usability of Reality DTx software and the AR glasses (Microsoft HoloLens 2 and Magic Leap 2); and (4) the future potential of Reality DTx in health care (Table 1). The interview guide was created by LESH and reviewed with MR and Strolll Limited (the manufacturer of Reality DTx) before and after pilot testing. The initial draft interview guide was updated with questions on willingness to continue the exercise program after trial, usability, commercial potential, and intended users. Researchers LESH and DJG conducted, respectively, 82% (n=18) and 18% (n=4) of the 22 interviews. The interviews were held from March to July 2023, were audio recorded and were transcribed verbatim by LESH. They lasted 57 (SD 14) minutes on average.

**Table 1:** Topics, subtopics, and operationalization of the semistructured interview guide.

Topics and subtopics		Operationalization
**Theoretical acceptability framework**		
	Affective attitude	“How did you feel about the Reality DTx^a^ training?”
	Perceived effectiveness	“To what extent did you experience effects from the training?”
	Intervention coherence	“Can you explain to me, in your own words, what the goal of the training was?”
	Self-efficacy	“How did you do in the training?”
	Opportunity costs	“To what extent did you have to give up on other activities to participate in the training?”
	Burden	“What did you think of the difficulty of the training (in general, not per game)?”
	Ethicality	“To what extent did the training fit your views on home-based gait-and-balance exercise?”
**FITT^b^** **principles**		
	Frequency of the training	“What did you think of the frequency of the training (ie, 5 days a week)?^c^”
	Intensity of the training	“What did you think of the intensity of the training?^c^” “Do you think your physical fitness has increased?”
	Type of AR^d^ exercises	“What did you think of the type of training (eg, gait and balance, type of exercises)?” “What game did you like the most?” “What game did you like the least?”
	Duration of the training	“What did you think of the duration of the training program as a whole (ie, 6 weeks)?^c^” “Would you have wanted to keep the device for longer?”
	Duration of 1 session	“What did you think of the duration of one training session (ie, 30 minutes)?^c^”
**Context-specific factors**		
	Training individually	“What did you think about training individually?”
	Training at home	“What did you think about training at home?”
	Scoring system	“What did you think about the scoring system that was used during training?”
	Remote supervision	“What did you think about remote supervision?”
**Usability**		
	Weight of the glasses	“What did you think about the weight of the AR glasses?”
	Field of view	“What did you think about the visibility of the holograms in the games?”
	Controlling the holograms	“What did you think about controlling the holograms or buttons with your hands? If you could chose, how would you like to control/select the buttons/holograms?”
	Wearer comfort	“How long do you think you could wear the AR glasses before it becomes uncomfortable? (...hour(s) ...min)”
	AR glasses	“What were your expectations about training with Reality DTx and how do you feel about them now?” “What did you think about the AR glasses?” “What would you change about the AR glasses?”
**Experiences during the last week of training** ^e^		
	Adverse events	“Did you experience any difficulties in terms of eye sight during the Reality DTx training? That is, were all letters and holograms clearly visible and readable?” “Did you experience any of the following problems this week: eye strain, dizziness, headache, something else, which is: ...?” “Did you fall/nearly fall during the training this week?”
	Technical issues	“Did you experience any technical difficulties this week?”
	Overall feeling of safety	“Did you feel unsafe at any point during the Reality DTx training?”
	Overall usefulness	“On a scale from 0 to 10, how useful did you find the training?”
	Overall user-friendliness	“On a scale from 0 to 10, how user friendly did you find the glasses/the Reality DTx app?”
**Future perspective**		
	Suggestions for improvements	“Do you have any suggestions for improving the Reality DTx training program?”
	Monthly subscription	“If you would need to pay a monthly prescription for Reality DTx (which includes the AR glasses, the application, and technical support), what would you pay?”
	Intended users	“What type of people do you think will benefit most from Reality DTx?”
	Future implementation in health care	“How would you feel if, in the future, Reality DTx would replace part of your supervised physical-therapy sessions with remotely prescribed home-based sessions resulting in less frequent in-clinic sessions, for example, once every 3 weeks instead of once a week?”

^a^Reality DTx: Reality Digital Therapeutics.

^b^FITT: frequency, intensity, type, and time.

^c^Participants were provided with multiple-choice answer options, such as “too infrequent—too often,” “too light—too hard” and “too easy—too hard,” to guide their responses. However, they were encouraged to explain their answers.

^d^AR: augmented reality.

^e^Data related to this topic were collected during the weekly telephone calls and used in the data analysis of the number of adverse events and the number of technical issues reported in the study by Hardeman et al [11].

### Analysis

We applied a reflexive thematic analysis as described by Braun and Clarke [31-33] in 6 phases, as presented in Table 2. We used an inductive, semantic approach that is, coding and theme development were directed by the content of the data and explicitly reflected the data. We aimed to remain close to what participants said while ensuring clarity and comprehension in naming and describing themes and subthemes. To ensure a critical realist approach, that is, to acknowledge the researcher’s role in the analytic process, while simultaneously ensuring that we remained close to the participants’ lived experiences, the research team maintained detailed reflexive memos. In this way, we could critically examine and document biases, assumptions, and the evolution of our themes. In addition, regular team triangulation sessions were held to enhance analytic rigor and to ensure that our interpretation remained grounded in the data (Table 2; phases 2-5). Transcripts and themes were analyzed and discussed in Dutch using Atlas.ti software (version 20; Lumivero). Themes and selected quotations were translated into English by LESH. Logs of the data analysis steps were kept.

**Table 2:** Description of the reflexive thematic analysis followed, including a description of the triangulation process, corresponding to the 6 phases [31-33].

Phase^a^	Description of the reflexive thematic analysis process as described by Braun and Clarke [31-33]^a^	Description of the reflexive thematic analysis data analysis process of this study
1. Familiarizing with data	Transcribing data (if necessary), reading and rereading the data, and noting down initial ideas	Data were transcribed by LESH. LESH and EvB familiarized themselves with the data.
2. Generating initial codes	Coding interesting features of the data in a systematic fashion across the entire dataset and collating data relevant to each code	Initial open codes of 4 transcripts were generated and thoroughly discussed and revised by LESH and EvB. Initial codes were collated in categories by LESH in a mind map that was presented, thoroughly discussed, and revised during multiple triangulation sessions between LESH, EvB, MvG, and EMH, while LESH open-coded the remaining data.
3. Searching for themes	Collating codes into potential themes and gathering all data relevant to each potential theme	Potential themes and subthemes, resulting from categories of open codes, were discussed in the first 5 triangulation sessions with the authors EvB, MvG and EMH and further refined by LESH.
4. Reviewing themes	Checking if the themes work in relation to the coded extracts (level 1) and the entire dataset (level 2) and generating a thematic “map” of the analysis	An initial thematic map was generated by LESH. Initial themes and subthemes were discussed in relation to the coded extracts, in relation to each other, and in relation to the entire dataset during the subsequent 4 triangulation sessions with the authors mentioned earlier.
5. Defining and naming themes	Ongoing analysis to refine the specifics of each theme and the overall story the analysis tells and generating clear definitions and names for each theme	Following the initial thematic map, the overall story was drafted and discussed between the authors mentioned earlier in the subsequent 4 triangulation sessions. Themes and subthemes were further defined and named until agreement was reached.
6. Producing the report	The final opportunity for analysis: selection of vivid, compelling extract examples; final analysis of selected extracts; relating back of the analysis to the research question and literature; and producing a scholarly report of the analysis	The drafted overall story was further refined, and corresponding selected extracts were replaced if they did not represent the theme or subtheme accurately.

^a^Adapted from the study by Braun and Clarke [31].

### Researcher Positionality

The analysis was conducted from a reflexive thematic analysis approach, informed by a critical realist orientation, while acknowledging that data interpretation is also shaped by the researchers’ disciplinary backgrounds and professional experience. While interpretation inevitably involved the researcher’s perspective, every effort was made to remain true to the participants’ own meanings and experiences, acknowledging the potential influence of the researcher’s subjectivity throughout the analytic process. The lead analyst (LESH) is a PhD candidate with a background in clinical psychology and neuropsychology who worked as a psychologist in rehabilitation. Her clinical experience contributed to a close reading of how participants described cognitive and emotional processes in everyday contexts. The analysis was supervised by EvB, a clinical epidemiologist and applied sciences lecturer with previous training and experience in qualitative interview methods. Their complementary perspectives, informed by neuropsychology and physical therapy, helped to frame the data in relation to rehabilitation goals, patient engagement, and perceived barriers. Triangulation sessions were held between LESH and EvB to support reflexivity and analytic consistency and were enriched by input from EMH and MvG, who brought in human movement science expertise. EMH is a PhD candidate and human movement scientist, and MvG is a PhD candidate specializing in fall prevention in the older adults as part of the In Balance trial [34], with experience in qualitative analysis. LESH, DJG, and EMH and author MR are part of the same research team at the Vrije Universiteit Amsterdam, with author JN involved as an external PhD cosupervisor of LESH. EvB and MvG are not part of the same team. Some of the participants had participated in previous trials and thus had a connection with researchers LESH, EMH, and DJG. LESH and EMH worked closely with all participants as part of the data collection of the Reality DTx feasibility trial, whereas DJG was only involved in the final stages of the data collection of the trial.

## Results

### Study Sample

The study included 22 people with Parkinson disease, 7 women (32%) and 15 (68%) men, with a mean age of 66 (range 51-82) years. The time since diagnosis was on average 8 (range 1-20) years, and participants were classified with a modified H&Y of 2 (14/22, 64%) or 2.5 (8/22, 36%). An overview of participant characteristics to characterize the sample is provided in Table 3. Medication remained stable over the waitlist and intervention period. For further participant characteristics, please refer to our feasibility results [11].

**Table 3:** Demographics and clinical characteristics of the sample assessed during baseline measurements (t0) of the feasibility study.

Participant number	Sex	Age (y)	Time since diagnosis (y)	Modified H&Y^a^	MoCA^b^ score	Total MDS-UPDRS^c^ score	PASE^d^ score
1	Male	72	1	2	26	34	182.3
2	Male	74	6	2	18	78	49.0
3	Female	60	2	2	27	39	238.9
4	Male	51	7	2.5	28	46	180.8
5^e^	Female	60	20	2.5	29	62	71.1
6	Female	68	8	2	28	59	40.0
7	Male	73	11	2	19	76	68.2
8	Female	56	1	2	29	70	93.0
9	Male	74	12	2.5	22	78	96.6
10	Male	59	6	2	25	53	89.9
11	Male	51	2	2	28	50	95.0
12	Male	65	6	2	27	68	211.8
13	Male	53	14	2	29	50	80.6
14	Female	57	13	2.5	28	78	78.7
15^e^	Female	66	11	2.5	30	67	180.0
16	Male	82	8	2.5	30	73	134.4
17	Male	74	5	2	29	78	160.6
18	Male	63	6	2	29	76	97.7
19	Male	78	4	2	26	68	171.0
20	Male	68	10	2.5	28	79	76.1
21	Male	71	15	2	30	51	151.4
22	Female	74	7	2.5	23	73	118.7

^a^Modified H&Y: the Modified Hoehn and Yahr Scale (ranging 1-5; following progression in symptoms).

^b^MoCA: Montreal Cognitive Assessment (total score is 30; a score of ≥26 is considered normal [35]).

^c^MDS-UPDRS: Movement Disorder Society-unified Parkinson’s Disease Rating Scale (ranging 0-272, higher total scores indicate more severe overall symptom severity).

^d^PASE: Physical Activity Scale for the Elderly (higher scores indicate higher physical activity levels).

^e^Participants who dropped out of the study due to medical circumstances unrelated to the study in weeks 4 and 6.

### Context

The sample can be further characterized according to a number of reported experiences, which is important for the interpretation of the findings. First, participants expressed a notable sense of responsibility to contribute to scientific research, which motivated them to adhere to the prescribed program to the best of their ability:

Because I had said “yes” once, I also wanted to follow through. To serve you as best as I can, I want to fulfil my commitment to the best of my ability.

Participant 19

Second, as reflected in the individual Physical Activity Scale for the Elderly and Montreal Cognitive Assessment scores (Table 3), participants showed variability in physical and cognitive abilities, with some being more physically active than others. This variability was also present in their expressions, for example:

And by then I have walked outside for four hours, half an hour in that sense, is more than easily met every day.

Participant 19

These participants who were relatively physically active were often more engaged in work or had busy retirement schedules:

Well, I was glad I had two days off every week, because honestly, having to train five out of seven days really kept me accountable, so to speak. But since I work full time and often trained in the evenings, it was like, okay, okay, let’s just get it done. I think if there hadn’t been any pressure, I might have skipped it more often.

Participant 3

Other participants had more severe physical or cognitive symptoms that interfered with their performance:

Well, first of all, he [partner] tells me: “Stand up straight, stretch your leg.” If he didn’t, I would forget. He helps me remember, so alone, I wouldn’t get anywhere.

In general, participants realized that their Parkinson symptoms would deteriorate over time rather than that they would improve, independent of their commitment to exercise in general:

Initially, I noticed a very positive effect. But lately, I feel like I’m just deteriorating because of the Parkinson.

Participant 5

Third, some participants expressed more experience and affinity with technology than others:

Since COVID, we’ve gotten used to doing everything remotely, so that works pretty well, although I do struggle sometimes because, you know, I’m not exactly a computer genius.

Participant 19

I know I’m very enthusiastic about these kinds of things, especially because it involves technology because I want to provide feedback from the user’s perspective to help improve it.

Participant 18

### Qualitative Findings

#### Overview

The results of the reflexive thematic analysis are described in 3 themes: “there was considerable interindividual variation,” “the program is complementary to supervised physical therapy,” and “adherence in the long term is crucial,” and 9 subthemes (Textbox 1).

**Themes and subthemes resulting from the reflexive thematic analysis.**

**There was considerable interindividual variation**

- Variation in perceived effectiveness
- Variation in experienced effort

**The program is complementary to supervised physical therapy**

- Supervision by the physical therapist
- Social connection
- Different treatment modalities within physical therapy

**Adherence in the long term is crucial**

- Flexibility
- Better visibility of progression in scores
- More variation in games
- The addition of a competitive element

#### There Was Considerable Interindividual Variation

There was considerable interindividual variation in perceived effectiveness and experienced effort.

##### Variation in Perceived Effectiveness

Participants identified various effects of the program. Examples included improvements in aspects of gait and balance—the program goals—as well as other improvements, such as increased endurance, decreased muscle rigidity, and increased confidence in their own body.

On one hand, some of the participants perceived specific improvements during gameplay:

Well, with turning, I would practice turning left or right since that’s my difficult side, with the boxing game. So I consciously focused on turning right, and I noticed it started to improve pretty quickly.

Participant 13

On the other hand, others described benefits that transferred to daily life activities, such as playing tennis:

Well, I was able to move more easily on the court again. I wasn’t constantly stuck or too late to get to the ball. Thoughts like: “Be careful or you’ll fall,” were much less present. I moved more easily with less freezing.

Participant 20

Yes, yes, I walk to the supermarket. I used to have to stop two or three times to catch my breath. But the last few times, I didn’t need to, I stopped once and continued.

Participant 6

Furthermore, some participants reported that the exercises required them to concentrate more, either on the task at hand or on the movement:

Other people too—people who want to move, not just their bodies but also their brain. Using their brains more, improving concentration—it’s good for that too...

Participant 6

Furthermore, some participants felt more cognitively alert or more physically active after the exercise sessions, although this distinction was not always clearly expressed:

Normally, you do things on autopilot. As a Parkinson patient, you have to think about it. After a training session, it’s physical, but it also feels like it wakes you up somehow...

Participant 12

Participants who did not perceive direct effects, characterized by relatively high levels of physical activity as described in the earlier context, offered several explanations. They suggested that their already high levels of physical activity may have diminished the potential gains of the intervention, that their gait and balance deficits were still relatively mild, that symptom decline over time masked the program effects, or that the duration of the intervention was too short to produce noticeable changes. Despite the absence of perceived effects, they expressed a strong belief that physical activity is inherently beneficial when diagnosed with Parkinson disease:

I also do physical therapy fitness, which is endurance already. I think it makes a difference depending on what stage of Parkinson’s you’re in and how active you can still be. If you don’t do much and then try these exercises, you’ll probably notice a difference. But if you’re already doing a lot, you might not see immediate effects. Still, I feel it’s worthwhile—I don’t feel like I’m wasting my time.

Participant 3

Some of these participants believed that the program was more suitable for people with Parkinson disease with more severe symptoms:

But as you go along, you notice that it really does something for you. If it could be developed into a fully functional system, I think it would have a lot of added value, especially for Parkinson patients with more severe problems. I don’t think I’m quite at that point yet.

Participant 12

The belief that physical activity is inherently beneficial when diagnosed with Parkinson disease aligns with the observation that some participants were not able to recite the program goal—to improve gait and balance—communicated at the start of the intervention. Instead, participants considered increasing physical activity as their primary program goal:

I initially struggled with it, but I believe the idea was to get people with Parkinson moving. You can prescribe exercises, and they’ll do them once, but the challenge is to do them more often. That’s why, having something that keeps you accountable—without it, it fades away.

Participant 16

##### Variation in Experienced Effort

Participants experienced the program as demanding for various reasons. Participants who were relatively physically active with busy work or retirement schedules, as described in the context earlier, experienced difficulty in integrating the program into their daily or weekly routines. They experienced considerable training load:

I must admit, I found it challenging to fit it into my schedule. That’s why I say it was demanding. After a whole day of activities, sitting down at 5 p.m, I didn’t always feel like doing an hour of training.

Participant 19

On the contrary, other participants expressed the desire to train more frequently than 5 times per week.

Participants with relatively lower activity levels and more severe physical or cognitive symptoms, as described in the context earlier (Table 3), experienced the program as more physically demanding:

I could do the training and complete it, but afterwards, I’d be really tired. I had to sit or lie down to regain energy.

Participant 6

Other remarks with regard to the experienced intensity of the program were that the exercise intensity was partially determined by the level of effort put into the exercises:

And that’s the big issue with this product—how enthusiastic will someone play the games? If I just play a game casually, it doesn’t have the same effect as when I tell myself: let’s go for it, I know it’s good for me and let’s challenge myself to keep improving each time.

Participant 18

#### The Program Is Complementary to Supervised Physical Therapy

##### Overview

During the interview, participants were presented with a hypothetical future scenario in which traditional supervised physical therapy would be replaced by a hybrid model that combines supervised physical therapy with the remotely prescribed Reality DTx gait and balance program at home. In this scenario, participants were given an example that illustrated this change, which would reduce the number of clinic visits and increase remotely prescribed exercise hours at home (Table 1 provides the scripted scenario). The discussion of this scenario resulted in the perspective that the program could complement, but not replace, supervised physical therapy sessions to a greater or lesser extent:

I personally think it doesn’t replace the physical therapist. I see it as an added value, an extra way of training...

Participant 8

Look, I do stability exercises, but I also do core exercises and such. So this can’t replace that, but you can certainly integrate some of your movements into it.

Participant 12

This perspective was shaped by several underlying factors, outlined in the subsequent sections in 3 subthemes: “supervision by the physical therapist,” “social connection,” and “different treatment modalities within physical therapy.”

As described earlier, some participants believed physical activity is inherently beneficial for Parkinson disease, even without perceived effects. In addition, they emphasized the importance of long-term adherence for effectiveness:

And the question is whether it sticks. I don’t think the chances are very high. At least, my personal theory is that it could help for a while, but I would have to keep doing these kinds of exercises.

Participant 20

On that note, participants emphasized the need for external control to sustain long-term adherence to the program. In the context of this study, researchers fulfilled this role by remotely prescribing and adjusting AR exercises in a shared decision-making process with participants through weekly calls to discuss their adherence, performance, and progress. These calls were perceived positively:

Well, you could modify the games and you asked whether everything was okay—I appreciated that.

Participant 9

##### Supervision by the Physical Therapist

In a future clinical scenario in which the program complements supervised physical therapy, some participants explicitly tasked the physical therapist with the remote supervision of adherence:

Look, if you go to the physical therapist, you’ll be exposed if you haven’t been practicing, so to speak.... You might need to provide some kind of access to the physical therapist for that.

Participant 8

Apart from adherence supervision, participants valued the physical therapist for providing real-time feedback on movement quality:

Yes, absolutely, it’s important that when you do it over a longer period, the physical therapist also looks at how you move and corrects you if you’re not doing it properly.

Participant 19

Participants emphasized the importance of executing the correct movement, but the level of trust in technology’s ability to elicit the correct movement varied. Some participants believed that the technology did elicit the correct movement:

So you have to stretch your arm far enough. So you make sure that you’re far enough from the pilar to punch. If you’re too close, it doesn’t work. The system’s requirements for the punch naturally lead you to a properly extended arm movement, so that’s beneficial for the exercise.

Participant 17

Whereas others were uncertain whether the technology had supported them sufficiently in executing the movements correctly at home:

You do perform the movement, but at the physical therapist, I try to make the movement so it’s really correct. With Hot Buttons, you don’t really know if you’re performing the movement optimally.

Participant 11

In line with the experienced uncertainty about the correctness of movements, the encouraging commentary provided by the AR glasses as a form of feedback was not perceived positively because it was not related to their actual performance:

Well, I want to be complimented because I actually did it really quickly, you know? Not because the software developer programmed a compliment every five minutes so that everyone gets the same “yay” and confetti, whether they’re fast or slow....

Participant 8

##### Social Connection

Some participants considered the AR program as complementary to rather than a full replacement of supervised physical therapy sessions because of the social aspect of group-based physical therapy. Many participants engaged in group-based physical therapy, supervised by their physical therapist. Participants described group-based physical therapy as a socially enriching activity, emphasizing connection with peers:

It could never replace it for me, especially the Parkinson-specific aspects of physical therapy. I mean, you can use equipment at any gym—that’s not the point. But talking about Parkinson and how to deal with it, how to turn over in bed, how to get on a bike properly—those very specific things can’t be replaced.

Participant 21

Group-based physical therapy was also identified as a motivator to exercise:

It’s a pretty lonely game—a game you play on your own. The actual motivation, in my opinion, is having a connection with others. That is more appealing to me.

Participant 17

##### Different Treatment Modalities Within Physical Therapy

Finally, some participants considered the AR program as complementary to rather than a full replacement of, supervised physical therapy sessions because physical therapists offer different treatment modalities that they deemed important. Examples are strength training, fall prevention exercises, and hands-on therapy:

Well, I go to physical therapy twice a week, and I’m in a group where I do many other things, so to speak. One day a week is for endurance, and the other day is for Parkinson exercises with the group—memory training, fall prevention, all kinds of things.

Participant 8

#### Adherence in the Long Term Is Crucial

##### Flexibility

If participants were asked to reflect on continuing with Reality DTx after study completion, they reported that the flexibility of the program was a very important aspect. Participants agreed that the program was particularly flexible, which supports exercise adherence in the long term. Several aspects of the program’s flexibility were highlighted, such as the speed of setting up the program, the efficient use of time of an exercise session, and the possibility to exercise each day, at the most convenient time, for example, when Parkinson symptoms were least disruptive, without having to travel. The perceptions of flexibility fit nicely with the daily fluctuations in symptoms that participants described:

An advantage is that it doesn’t matter much at what time you do it specifically. You can decide for yourself, now I feel good, let’s do it now because it is least inconvenient.

Participant 18

A couple of improvements for long-term adherence to the program were suggested, outlined in the following subthemes: “better visibility of progression in scores,” “more variation in games,” and “the addition of a competitive element.”

##### Better Visibility of Progression in Scores

An important improvement relates to the scoring system. The score was presented at the end of each game; however, without comparison to previous scores (ie, only a podium of personal best scores was presented as a reference). Participants perceived the score as a very important motivator. They expressed the desire to see their scores improve, to become better at a game:

You need to find motivation somewhere. You can try to be faster because you want to score more points. Even though you know it’s only about the movement, you still want to get as many balls in the net as possible. That’s just human nature.

Participant 17

Yes, but I do think, one of the motivations for me was being able to improve myself through the game. You don’t really win, but it’s still a kind of victory over yourself. You think, “Yes, it’s higher, I did it!”

Participant 3

With the score as an important motivator, participants indicated the importance of having a clear overview of their scores and a sufficient understanding of how to improve them. They wished to track their progression over time.

Participants also discussed the desire to feel challenged, which was also primarily related to the possibility improving scores:

Well, I think when you reach a higher level, it becomes harder to beat your record. That’s a bit demotivating because maybe you do better with 24 puzzle pieces instead of 12, but you don’t see it. I find that disappointing.

Participant 3

On a final note, technical issues encountered during the study sometimes hindered participants from continuing an exercise session, which negatively impacted the ability to improve their scores. Looking ahead, the stability of the technology was identified as an important area for improvement.

##### More Variation in Games

To adhere to the program in the long term, participants indicated a desire for a broader selection of games to prevent boredom:

But I think if there’s a continuous stream of new content, it keeps things fresh....

Participant 12

More variety within a game was also mentioned as a way to increase the level of challenge of a game. Suggestions to increase the level of challenge or for new games were to incorporate a cognitive element or to increase the variety of movements or stimuli. The level of challenge was one of the primary factors influencing participants’ selection of the most enjoyable game. Another factor was the recognizability of games related to sports they had played in the past, such as tennis:

Well, the boxing game, the turning—it’s part of the exercise, turning and turning. So it’s an exercise too, but at some point, you get a bit fed up with it.

Participant 16

##### The Addition of a Competitive Element

Although participants felt that the program could not replace the sense of connection that group-based physical therapy provides, they suggested implementing a competitive element into the program. Some suggested including a virtual ranking with other users, and others suggested incorporating a multiplayer option in the same (virtual) environment:

I’ve wondered if digital games or digital challenges could motivate you. I mean, like what I do with my physical therapy group—a few men who cheer each other on with great enthusiasm. That might be motivating....

Participant 17

## Discussion

### Principal Findings

This qualitative study aimed to explore the acceptability of Reality DTx, a home-based gamified gait and balance exercise program for people with Parkinson disease. The findings reveal considerable interindividual variation in perceived effectiveness and experienced effort, which corresponded to differences in physical activity, physical ability, and cognitive ability observed in our sample (Table 3). Still, participants consistently expressed a strong belief that physical activity is inherently beneficial when having Parkinson disease. They recognized the added value of the Reality DTx program as a complement to supervised physical therapy sessions. Participants also expressed that Reality DTx cannot fully replace supervised physical therapy sessions because of their desire for external control of the clinician to encourage long-term adherence and provide meaningful feedback on movement quality, as well as their desire for social connection inherent to group-based physical therapy and other treatment modalities offered by the physical therapist not being part of Reality DTx. An important element that was valued by the participants was the flexibility of the program, supporting long-term exercise adherence. The program was considered flexible in both time (eg, exercising when convenient in terms of symptom fluctuations and daily routines) and place (eg, exercising in your living environment rather than in a clinic, without having to travel). Participants suggested that adherence in the longer term would benefit from better visibility of progression in scores, more variation in games, and the addition of a competitive element.

### The Indispensable Role of the Clinician

It is important to interpret our findings within the context of the research setting, where researchers remotely prescribed and supervised the Reality DTx program [11]. This deviates from real-world scenarios, where clinicians will typically do this, probably taking a hybrid therapy approach combining remotely prescribed independent exercise at home with supervised physical therapy in the clinic (which aligns with “care as usual” in this study design). The absence of expert supervision in this study might have affected participants’ ability to envision the suggested partial replacement of supervised therapy sessions by Reality DTx sessions at home, which would result in a reduction of clinic visits—a future scenario that might be inevitable in light of diminishing accessibility to health care [3-6]. The importance of expert supervision was supported by reported needs for external control to encourage long-term adherence and to provide meaningful feedback on movement quality and a better understanding of the program and exercise goals, all known as important elements of (remote) physical therapy [17,20,27,36-39]. Certainly, the clinician—and by extension the clinical relationship—is indispensable in the future use case of remotely prescribed and supervised Reality DTx. Patients and clinicians describe this relationship as one initially built on face-to-face interaction that encompasses a level of trust, positive regard, and knowledge of the patient and illness [40].

We acknowledged the value of this relationship in a follow-up clinical trial where the Reality DTx program starts supervised in the clinic, building a relationship between patient, therapist, and technology, before continuing to exercise independently at home, complementary to usual care. Exercises are remotely prescribed and supervised by the clinician [41]. Technology could support the clinical relationship even further by providing meaningful feedback on movement quality to both the patient and the clinician (enhancing knowledge of performance [40] and patient self-efficacy in terms of the TFA [16]), which also aids motor learning [42]. For example, feedback on movement quality could be generated with motor parameters derived from the AR data during gameplay, such as step length [43-45]. These AR-based motor parameters and other variables, such as physical activity levels and disease severity, could also be used to support the clinician in intervention personalization in terms of optimal training dosage, challenge, and difficulty progression [46,47]. Given the value of the clinical relationship, the clinician should play an active role in this process [31,33].

### Other Promoters of Long-Term Exercise Adherence

Besides remote supervision of adherence and meaningful feedback on movement quality, the flexibility of the program was an important acknowledged characteristic of the program to support long-term exercise adherence. The possibility to exercise at home could potentially increase the number of exercise hours, as it overcomes often-reported barriers such as travel time to the clinic and fluctuations in motor symptoms that affect motor performance during exercise [48]. Furthermore, social connection to peers, experienced as a missing element by some participants, has consistently been recognized as a key motivator for long-term exercise adherence in previous Parkinson disease research [21,24,36-38]. Moreover, the finding aligns with the psychological need for relatedness as one of the underlying factors of the well-established self-determination theory of intrinsic motivation [49]. On the contrary, group-therapy has also been reported as confrontational, that is, to be confronted with motor disabilities of oneself or peers [36,38]. In that regard, home-based exercise such as Reality DTx might be a suitable alternative. An element of competition, which could be added to the Reality DTx software, could provide a sense of relatedness if desired.

### Putting Reported Experiences in Perspective

Although participants did not envision a partial replacement of supervised physical therapy sessions, the allocation of Parkinson care to the home environment might be inevitable in light of the growing demand and diminishing capacity of the health care system. Hybrid and technology-enhanced interventions, such as Reality DTx, are needed to lower the burden on health care professionals and to foster self-management of people with Parkinson disease [3-6]. However, the implementation of technology-enhanced interventions such as Reality DTx is rather complex because of multiple dynamically and unpredictably interacting domains, such as the domains of the innovation recipients, innovation deliverers, and key decision-makers [12,13,50]. As for the domain of the practice owners, as key decision-makers, concerns related to technology-enhanced interventions have been raised, such as a lack of logistic support setting up the technology and increased administrative workload and costs [24]. With regard to the wider system of key decision-makers, financial and regulatory bodies may not support technology-enhanced interventions [12]. Motivations, values, and norms of the involved stakeholders at each domain and across domains should be carefully weighted to explore and reduce or “run with” complexity [13,50]. This qualitative study will provide these other stakeholders with the perspective of the innovation recipients, people with Parkinson disease. We have informed our public-private partner, Strolll Limited, about the research findings (including the items from Table 1 that were not reported in this paper, such as usability) for further development of Reality DTx.

### Strengths, Limitations, and Future Research

To the best of our knowledge, this is the first study into AR home-based exercise for people with Parkinson disease that adopts a qualitative approach and explores the direct subjective experiences of people with Parkinson disease. This is a strength, because these direct experiences complement the more indirect measures of acceptability of the feasibility trial [10,11], such as adherence rates, and provide insights into the mechanisms of change of Reality DTx for gait and balance exercises at home. For example, external control of the intervention by a clinician to encourage long-term adherence and provide meaningful movement and game performance feedback, and the flexibility of the program, appear to be important mechanisms of change in maintaining the relatively high adherence rates and improving motor performance.

Another strength of this study is the early involvement of the intervention recipients in this preimplementation phase [6,18]. The needs of people with Parkinson disease reflected in the findings will inform future Reality DTx development and implementation. In addition, the findings will also inform the intervention deliverers, that is, the clinicians, as we aim to explore the acceptability among clinicians and the appropriateness of the program as part of clinical practice in the pragmatic clinical trial mentioned earlier [41]. The trustworthiness of this study was warranted with extensive triangulation with internal and external researchers who brought various perspectives to the table and reflected extensively on how these perspectives affect data analysis [51]. Furthermore, the steps of the reflexive thematic analysis were followed in an iterative and rigorous manner, reviewing open coding and themes and subthemes, and data analysis steps were recorded and reported. The elaborate description of our sample and context aids the understanding of the transferability of the results. These methodological study strengths notwithstanding, there were also a number of methodological limitations to point out. First, one researcher (LESH) in the triangulation was also involved in the feasibility trial and conducted most of the interviews. Ideally, to increase the trustworthiness of the findings, an independent interviewer could be considered in the future [30,51]. Second, the intervention was conducted in a research setting, which is not only a different context but also one that may have introduced a number of limitations, such as a gratitude bias (ie, people with Parkinson disease may have felt compelled to adhere to the exercise program out of a sense of duty to the research, see our remarks in the Results section context), recruitment bias (ie, recruited participants might have been more interested in technology so-called early adopters [52] compared to the general population of people with Parkinson disease, see our remarks in the Results section context) and responder bias (ie, 2 participants who dropped out of the feasibility study were not interviewed, potentially missing valuable insights or opposing perspectives that could challenge the study’s findings).

These biases and the broadening of recruitment strategies in terms of clinical characteristics, such as symptom severity (modified H&Y 2-2.5 in this trial), should be addressed in future research to improve the generalizability of acceptability results. For example, purposive sampling of people with Parkinson disease with various user characteristics (eg, different levels of affinity with technology) may reduce recruitment bias related to early adopters. Furthermore, symptom severity and physical activity level are suggested to affect the intervention’s acceptability in terms of perceived effectiveness and experienced effort (burden, in terms of the TFA [16]) and should be addressed in further development of the intervention to improve acceptability and explore clinical implications. A potential clinical implication of these findings, in particular, could be that the intervention is more appropriate and easier adopted by people with Parkinson disease with lower physical activity levels, as suggested in a previous study [53]. Acceptability could be improved for people with Parkinson disease with various physical activity levels and disease severities through adjustable intensity levels and personalization based on AR motor parameters [43,45-47]. The impact of individual differences in clinical (eg, physical activity level and symptom severity) and user (eg, affinity with technology) characteristics on the acceptability and adoption of the intervention should be explored in future effectiveness-implementation trials [22].

### Conclusions

People with Parkinson disease (H&Y 2-2.5) considered Reality DTx acceptable as an exercise program complementary to supervised physical therapy. Mechanisms of change for maintaining high adherence rates and a progressive-but-achievable intervention and for improving motor performance were identified and included external control of the intervention by a clinician, meaningful movement and game performance feedback, and the flexibility of the program. Individual differences in physical activity levels and disease severity may further explain acceptability in terms of perceived effectiveness and experienced effort. In light of the growing demands on health care, replacing supervised physical therapy sessions in the clinic with remotely prescribed therapy at home might be inevitable. In anticipation of this transformation, Reality DTx could better fit the needs of people with Parkinson disease and support long-term adherence to exercise by improving real-time movement feedback, by enhancing the visibility of progression in scores, by increasing variation in games, and by incorporating a competitive element into the game. Additional qualitative, in-depth research and weighting of interests, values, and norms among stakeholders in the wider system of users is needed to arrive at an encompassing evaluation of the acceptability of Reality DTx or related interventions.

## Abbreviations

AR: augmented reality
COREQ: Consolidated Criteria for Reporting Qualitative Research
H&Y: Hoehn and Yahr
Reality DTx: Reality Digital Therapeutics
t0: baseline
t1: before intervention
t2: after intervention
TFA: theoretical framework of acceptability
